# Three decades of neuroscience research using animal models of ADHD and ASD: a bibliometric analysis

**DOI:** 10.3389/fpsyt.2025.1528205

**Published:** 2025-05-19

**Authors:** Godfried Dougnon, Hideaki Matsui

**Affiliations:** Department of Neuroscience of Disease, Brain Research Institute, Niigata University, Niigata, Japan

**Keywords:** ADHD, autism spectrum disorders, animal model, neurodevelopmental disorder, trends, bibliometric

## Abstract

**Introduction:**

Attention-deficit/hyperactivity disorder (ADHD) and autism spectrum disorders (ASD) are two increasingly prevalent neurodevelopmental disorders (NDDs), often accompanied by significant daily-life challenges. Animal models play a crucial role in studying these conditions, and recent advances have highlighted the potential of animal models such as mice, rat, zebrafish, *Drosophila* or *Caenorhabditis elegans* for investigating NDDs. However, despite growing interest, a complete understanding of these disorders has yet to be achieved. We believe that to properly address these NDDs, it is important to analyze the heterogeneity of ADHD and ASD research.

**Methods:**

This study comprehensively analyzes ADHD and ASD-related scientific publications from January 1990 to December 2023 using data from the Web of Science (WoS), exploring trends in global research output, impact factors, citation metrics, the predominant use of animal models, the contribution of major countries and funding information.

**Results:**

Out of the 10,844 papers from WoS, we curated 5,883 papers and identify mice and rat as the primarily used animal models, and a progressive use of zebrafish, *Drosophila* and *C. elegans* since the early 2000s. The countries conducting research on ADHD and ASD were principally the United States (3,059 articles), followed by China (487 articles), the United Kingdom (459 articles), Japan (440 articles), Germany (413 articles). We further show that impact factors and journal citations were relatively similar among the major publishing countries. Interestingly, key research funders were the National Institute of Health (NIH), the National Institute of Mental Health (NIMH), and the Japanese Ministry of Education Culture Sports Science and Technology (MEXT), making important contributions to their respective countries’ publications. Of note, Africa and Oceania have a lower volume of publication; however, our network analysis indicates a recent peak in research interest and ADHD/ASD awareness in some countries like Ghana or Portugal.

**Conclusion:**

The findings highlight significant advancements and collaborative efforts in ADHD and ASD research over the last three decades, underscoring the importance of international cooperation in addressing these complex neurodevelopmental disorders.

## 1 Introduction

Attention-deficit/hyperactivity disorder (ADHD) and autism spectrum disorder (ASD) are among the most prevalent neurodevelopmental disorders (NDDs), affecting millions of individuals worldwide. ADHD is characterized by persistent patterns of inattention and/or hyperactivity/impulsivity, significantly impacting academic performance, occupational functioning, and social relationships (1–3). ASD encompasses challenges in social interaction, communication, and the presence of repetitive behaviors and restricted interests. The global prevalence of ADHD is estimated to be between 5-7%, while ASD affects approximately 1% of the population (2, 4–6). However, recent evidence suggests that the prevalence of ADHD in people with ASD ranges from 50 to 70% (7, 8).

Given the substantial burden these disorders place on individuals, families, and healthcare systems, extensive research efforts have been directed toward understanding their etiology, pathophysiology, and treatment. Animal models play a crucial role in this research by allowing for controlled experimental manipulation and observation of behaviors and biological processes relevant to ADHD and ASD. Commonly used models include the house mouse (*Mus musculus*), the brown rat (*Rattus norvegicus*), the zebrafish (*Danio rerio*), the common fruit fly (*Drosophila melanogaster*), and the roundworm (*Caenorhabditis elegans*), each offering unique advantages in studying the genetic, molecular, and behavioral underpinnings of these disorders (9, 10). In our previous report (11), we reviewed the mouse and zebrafish models of ADHD and ASD, highlighting their various advantages for understanding these disorders. For instance, zebrafish models allow for high-throughput behavioral screening and real-time imaging of neuronal activity, while mouse models provide robust genetic tools and well-established behavioral paradigms that closely mimic human symptoms. These complementary approaches enhance our ability to investigate the molecular and behavioral mechanisms underlying ADHD and ASD (12, 13).

ADHD and ASD may be influenced by factors such as social deprivation, genetic and metabolic diseases, immune disorders, infectious diseases, nutritional factors, physical trauma, or toxic and environmental factors (14). It is critical to analyze the progress of scientific research using animal models of diseases, not only to identify patterns, but also to have a clear understanding of what has been done and what remains to be done. In recent years, scientific publication database mining tools have been utilized to estimate specific research outcomes by measuring publication trends (15, 16). As research using animal models of ADHD and ASD is increasing, we here aim to provide a comprehensive analysis of ADHD and ASD-related publications from January 1990 to December 2023. Accordingly, we manually investigated each of 5,883 Web of Science (WoS)-listed ADHD/ASD publications and extracted key metrics, including author affiliations, areas of neuroscience, journals, citations, funding information, the use of different animal models, and the contributions of various countries, to identify key areas of focus and collaboration in the field. Additionally, we investigate whether research activity correlates with the prevalence of these disorders in the major ADHD/ASD-related research-performing countries (the United States, the United Kingdom, Japan, China, France, Italy, Canada and Germany). This analysis will help highlight the progress made in understanding ADHD and ASD using animal models over the last three decades and identify specific points where further research is needed.

## 2 Methodology

**Figure 1:**
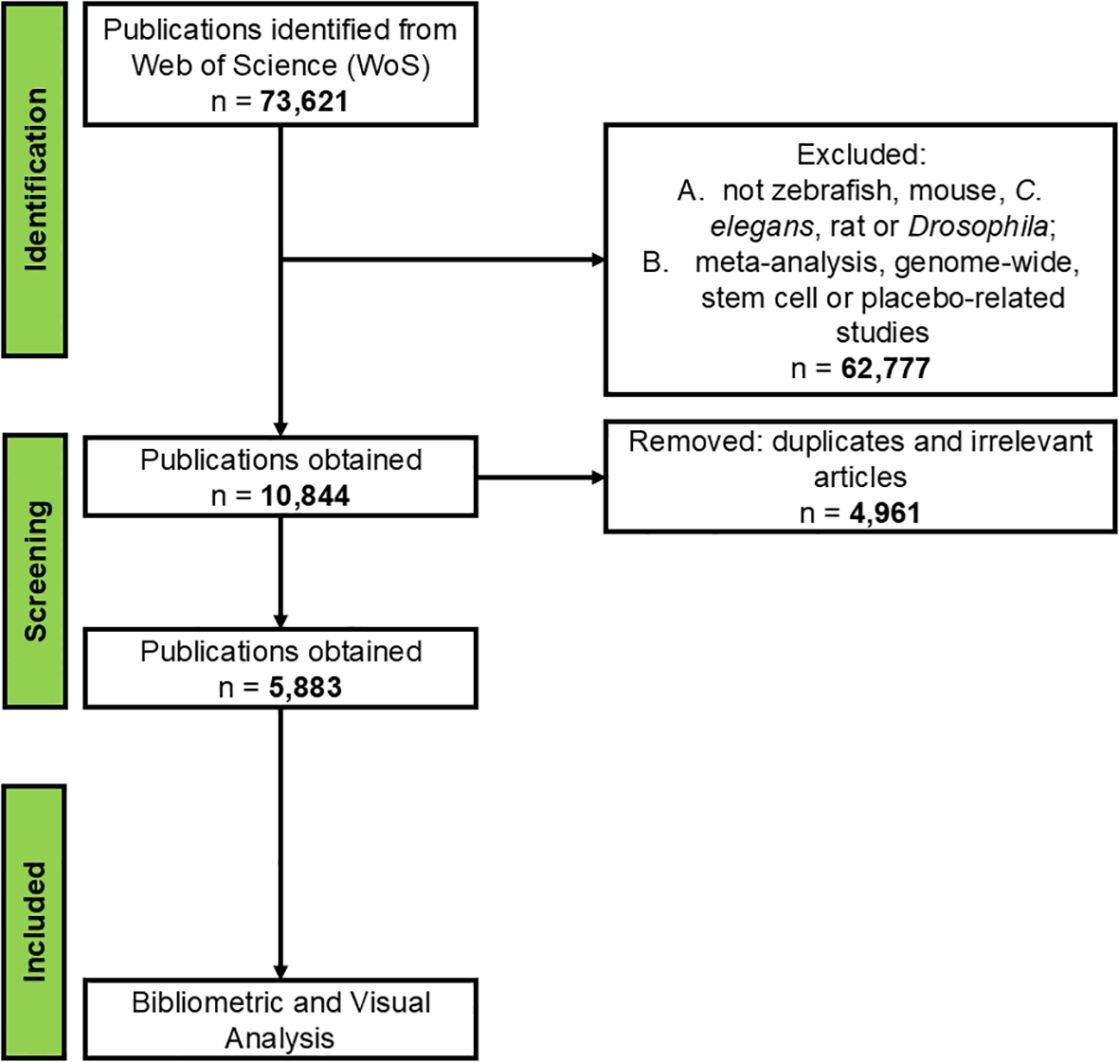
Flowchart for data cleaning and selection process based on PRISMA.

The steps involved in the bibliometric analysis (see **Figure 1** based on PRISMA guidelines) included data collection and search strategy, cleaning, analysis, representation of data in the form of figures and tables, and discussion and interpretation of the results. PRISMA stands for Preferred Reporting Items for Systematic Reviews and Meta-Analyses, which provides a structured framework for reporting the bibliometric analysis process by ensuring clarity, transparency, reproducibility, rigor, and comprehensiveness of the bibliometric study (17).

### 2.1 Data collection and search strategy

We collected data from the WoS database (18), covering publications from January 1990 to December 2023. Our search strategy combined terms related to ADHD (“ADHD”, “attention deficit hyperactivity disorder”, “attention deficit disorder”) and ASD (“autism”, “autism spectrum disorders”), with terms referring to animal model terms (“mouse”, “rat”, “zebrafish”, “*Drosophila*”, “*C. elegans*”). The search was conducted using Boolean operators as follows: (((((ALL=(adhd)) OR ALL=(attention deficit hyperactivity disorder)) OR ALL=(autism)) OR ALL=(autism spectrum disorders)) OR ALL=(attention deficit disorder)) NOT ALL=(review) and Article (Document Types) and English (Languages) AND ALL=((zebrafish) OR (mouse) OR (*C. elegans*) OR (*drosophila*) OR (rat)) NOT ALL=((meta-analysis) OR (genome-wide) OR (stem cell) OR (placebo)).

### 2.2 Inclusion and exclusion criteria

We included only original research papers written in English. Reviews, meta-analysis, genome-wide, stem cell or placebo studies were excluded. After performing the initial search, we manually removed duplicates and irrelevant articles, resulting in a total of 5,883 potential papers (**Supplementary Table 1**). Irrelevant articles included those that used models unrelated to mice, rat, zebrafish, *Drosophila* or *C. elegans*, or investigated unrelated neurological disorders. To identify major contributing countries, we focused on countries with at least 250 publications within the specified time period. This cutoff was chosen based on preliminary data assessment, enabling a more focused and manageable examination of key contributors. As a result, 8 major contributors were identified: the United States, the United Kingdom, Japan, China, France, Italy, Canada, and Germany.

### 2.3 Data processing and classification of countries/continents

During our search strategy, WoS provides records in Medline of MeSH terms. Using Microsoft Excel, data obtained from the merged WoS + Medline output files were manually curated. Authors’ affiliations were used to identify contributing countries and identify collaborations all over the world. For practicability, WoS automatically provides the total number of citations and the publishing journals. Impact factors in 2023 were retrieved from Clarivate Analytics or from Scimago. Furthermore, WoS also provides details on research areas, and we collected these data for each country relevant to this study. Briefly, research areas investigating ADHD and ASD included, for example, neuroscience, behavioral science, and psychiatry. Additionally, each paper was attributed to a research topic, based on WoS citation topics classification. Specifically, topics included, for example, ASD and neurodevelopmental disorders, neuroscience, psychiatry, sleep sciences and circadian systems, epilepsy and seizures, etc. Data on GDP, population, and life expectancy were retrieved from Worldometers (https://www.worldometers.info/gdp/gdp-by-country/).

To map and analyze global contributions to ADHD and ASD research, we classified countries and continents using the United Nations (UN) Geoscheme classification system from the United Nations Statistics Division (UNSD), Country and area codes (M49) (https://unstats.un.org/unsd/methodology/m49/). This system categorizes countries into defined regions and subregions, ensuring consistency and minimizing potential biases in classification.

### 2.4 Map generation and visualization

Maps were generated using Datawrapper (https://app.datawrapper.de/), which allowed for the visualization of global distribution patterns, including the number of publications, international collaborations, and research area contributions. Geospatial data were imported, and countries were color-coded according to their respective publication counts and collaborative networks. For consistency, countries were assigned to continents and regions based on the UN Geoscheme, as follows: Americas (North America, Central America, and South America); Europe (Eastern, Western, Northern, and Southern Europe); Asia (Eastern, Southern, South-Eastern, and Western Asia); Africa (Northern, Sub-Saharan, and Southern Africa); Oceania (Australia, New Zealand, and Pacific Islands).

### 2.5 Data analysis

Data analysis was performed using Microsoft Excel (.csv) files saved from WoS, GraphPad Prism version 9, and custom-written Python codes. The analysis focused on different key aspects such as: publication trends (the number of publications per year and the use of different animal models were analyzed to identify trends over time); geographic distribution (publications were categorized by continent and country, with a focus on the top eight countries in terms of research output); impact factors and citations (journals were categorized based on their impact factors, and citation metrics were analyzed to assess the influence of research from different countries); research focus areas (the top 10 research areas, as classified by WoS, were identified for each country to understand the primary focus areas within ADHD and ASD research); micro-topics (specific micro-topics within the publications were identified and analyzed to highlight detailed areas of investigation); funding sources (the analysis included identifying the primary funding sources for ADHD and ASD research, with a focal point on the United States (principal contributor in ADHD and ASD research) and international collaborations). Finally, to assess international collaborations, we analyzed co-authorship networks by tracking institutional affiliations across contributing countries, average citations and publication trends over time. Each paper’s corresponding affiliations were mapped to visualize collaborative efforts among regions. Network analysis was conducted using VOS viewer software version 1.6.20 (Leiden University, https://www.vosviewer.com). VOS viewer allows easy visualization of nodes and their various relationships, identified as citation or publication strength (19, 20). Publication strength refers to the number of publications associated with a particular node (e.g., a country or research topic) within the network, which reflects the relative research output. This metric was used to assess the prominence of certain nodes in the visualized network.

All retrieved papers were used to construct **Figures 2**, **3A**, and **Supplementary Figures 1**-**5**, **9**-**12**, while a detailed analysis was conducted on the top 8 countries, each with a minimum of 250 publications, for the generation of **Figures 3B–D**, **Figures 4**–**8** and **Supplementary Figures 6**-**8**.

**Figure 2:**
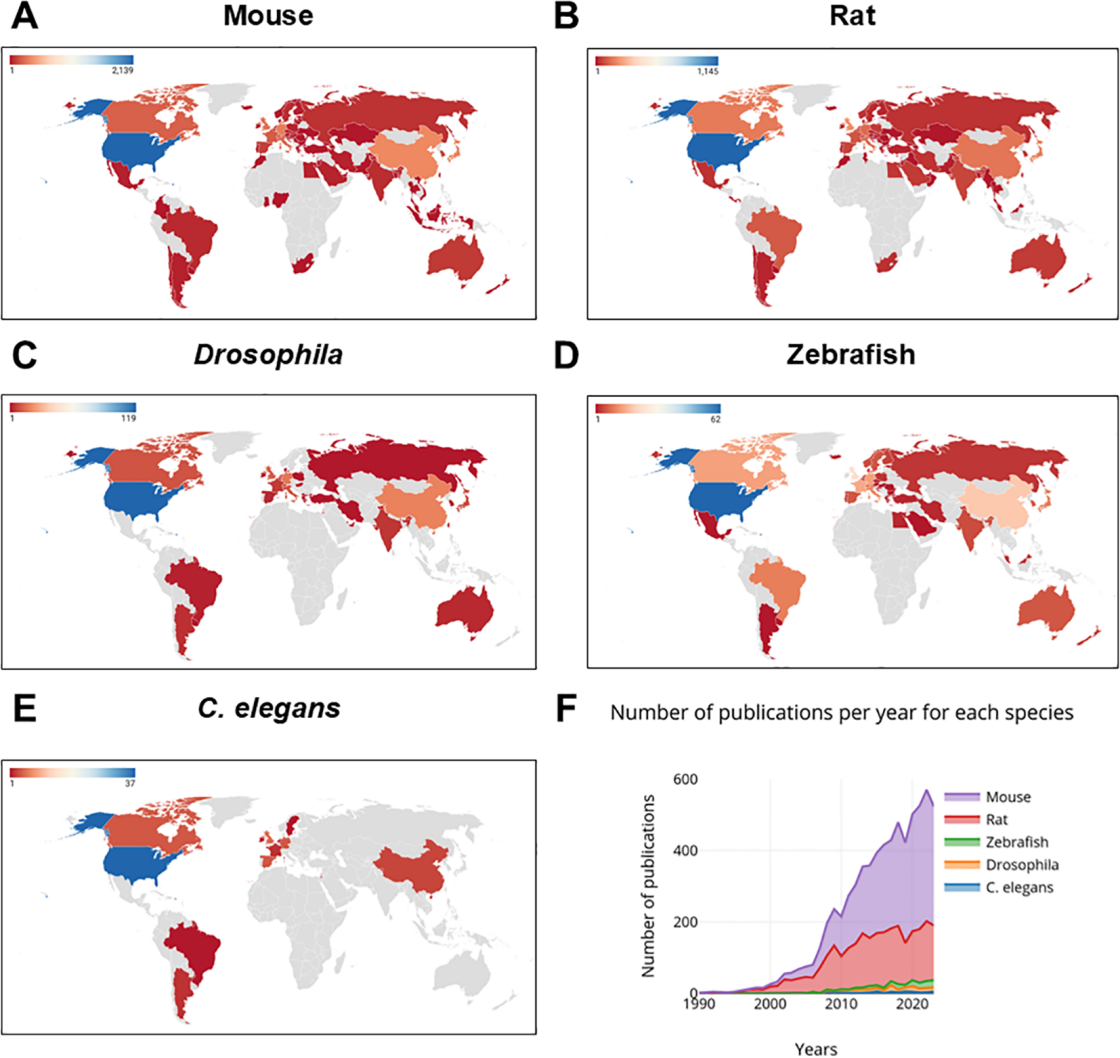
Worldwide ASD and ADHD publications from 1990-2023. **(A)** Overview of publications using mouse. **(B)** Overview of publications using rat. **(C)** Overview of publications using *Drosophila*. **(D)** Overview of publications using zebrafish. **(E)** Overview of publications using *C*. *elegans*. **(F)** Number of publications per year for each species (N = 3886 for mouse, 2544 for rat, 167 for *Drosophila*, 148 for zebrafish and 52 for *C*. *elegans*). Maps were created using Datawrapper (https://app.datawrapper.de/).

**Figure 3:**
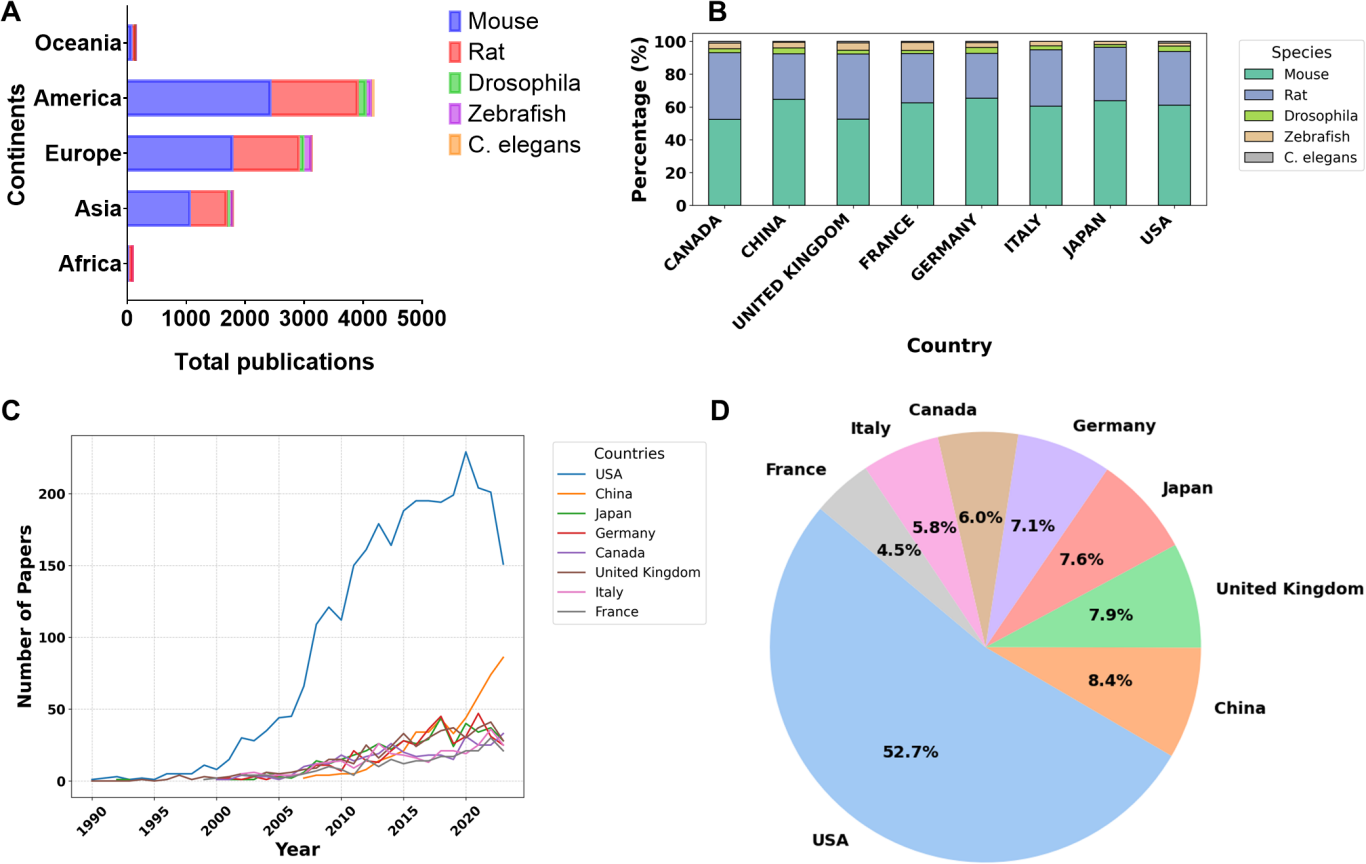
ADHD/ASD publications in the world from 1990-2023. **(A)** Total number of publications per species for each continent (Africa, Asia, Europe, America and Oceania). **(B)** Percentage of publications per species for the 8 main countries contributing to ADHD/ASD research. **(C)** Number of papers published annually by the top 8 countries from 1990 to 2023. **(D)** Percentage of ADHD/ASD publications per country.

**Figure 4:**
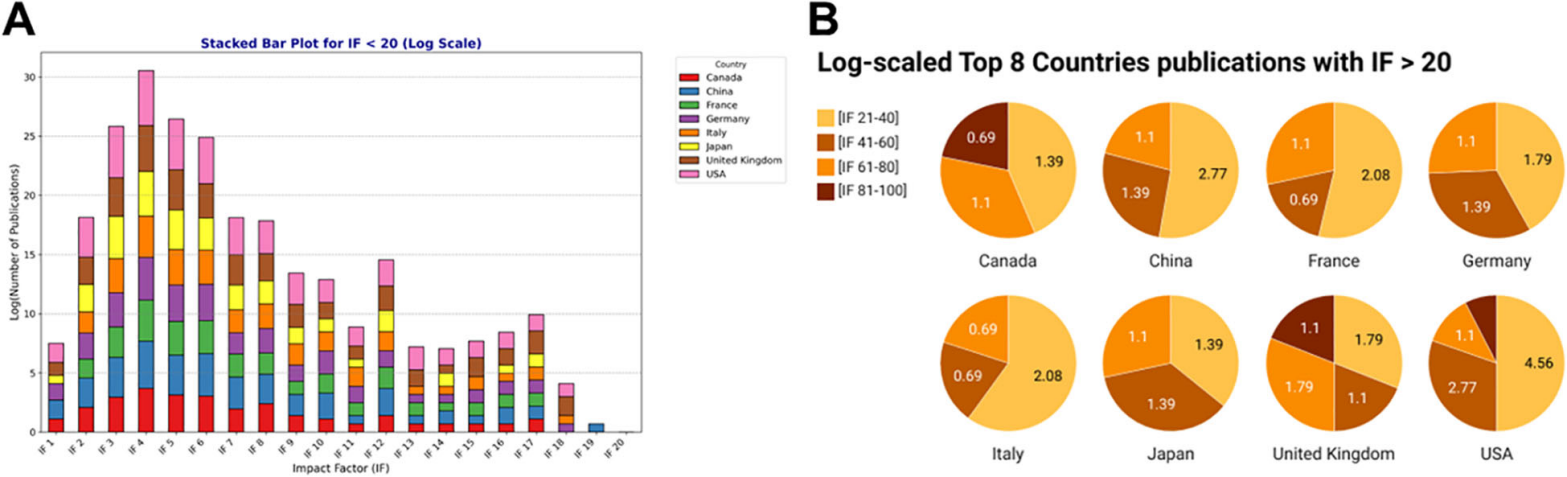
Impact factors for the major contributing countries. **(A)** Stacked bar showing the top 8 countries publications with impact factors (IF) < 20. Values represent log scales of the number of publications (*y*-axis) by the impact factors (*x*-axis). **(B)** Pie charts showing the top 8 countries publications with IF > 20. Values in the pie charts represent the log scales of number of publications for each range. To allow easy visualization, IF are ranged in groups of [21-40], [41-60], [61-80] and [81-100]. Created with Datawrapper.

**Figure 5:**
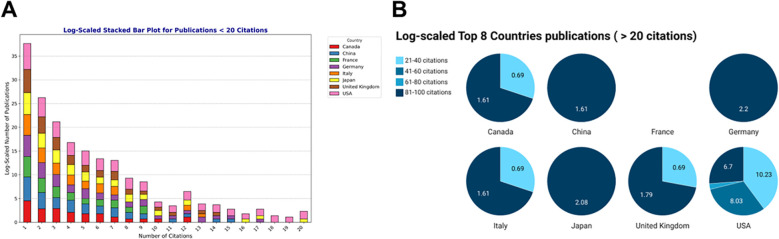
Publication citations for the major contributing countries. **(A)** Stacked bar showing the top 8 countries publications with number of citations ranging < 20. Values are log scales of the number of publications (*y*-axis) by the number of times cited (*x*-axis). **(B)** Pie charts showing the top 8 countries publications with number of citations > 20. Values in the pie chart are the log scales of number of publications for each range. To allow easy visualization, number of citations are ranged in groups of [21-40], [41-60], [61-80] and [81-100] citations. Created with Datawrapper.

**Figure 6:**
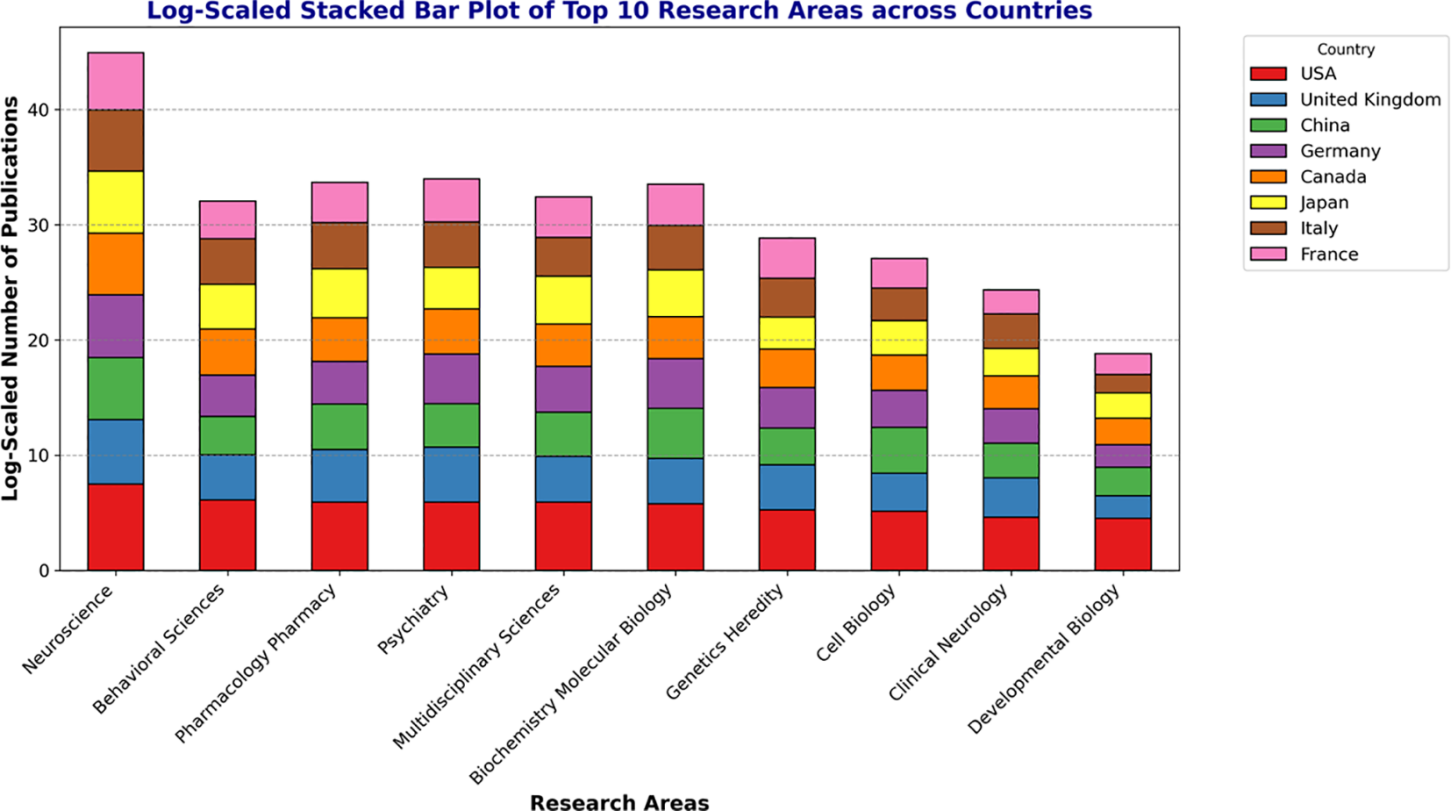
Research areas. The graph represents a log-scaled stacked bar of the top research areas regarding ADHD/ASD research for the top 8 contributing countries (N = 10 research areas).

**Figure 7:**
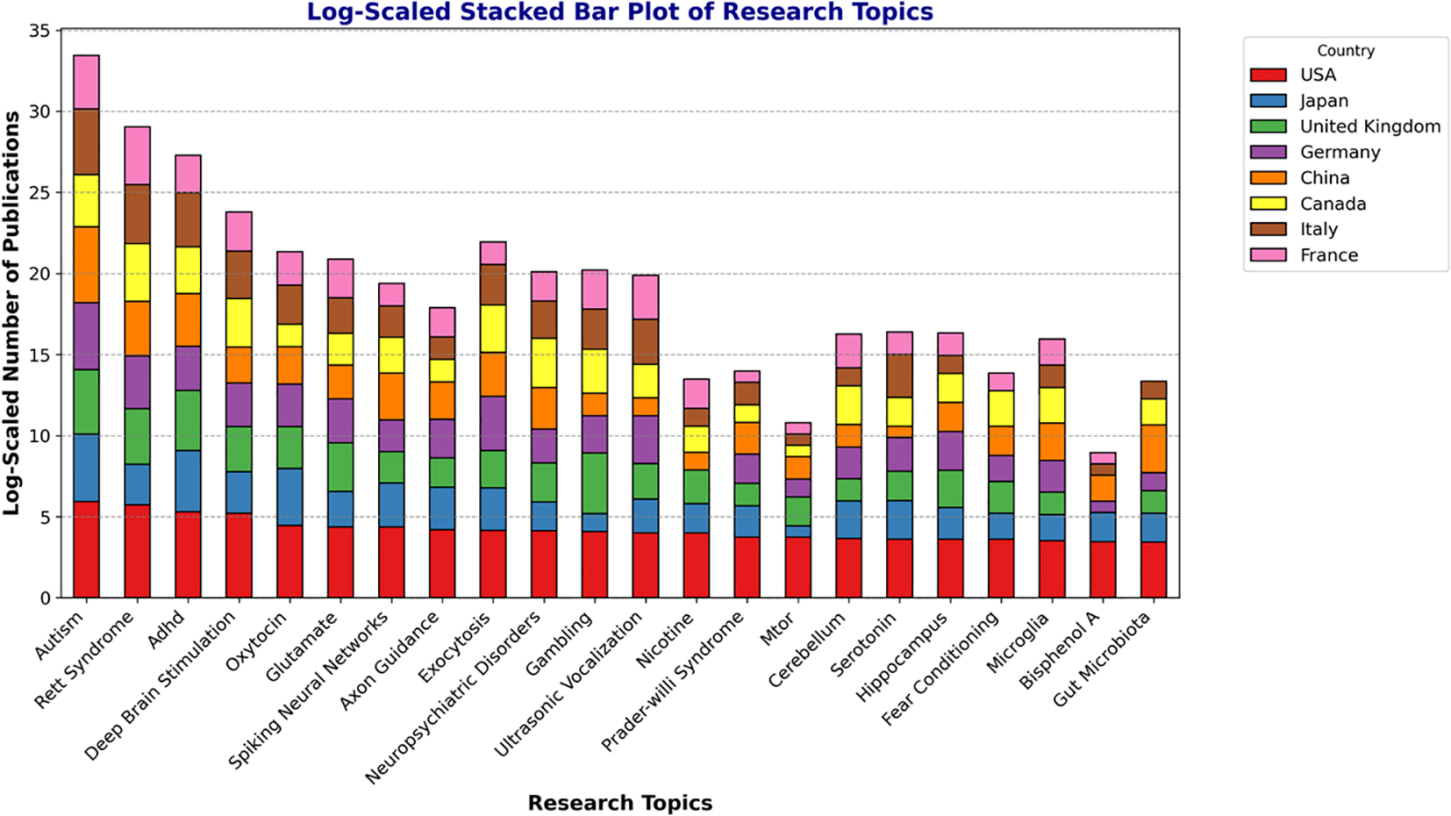
Micro-topics investigated for ADHD/ASD research. The graph shows a log-scaled stacked bar of the micro-topics represented by ADHD/ASD research for each country (N = 22 topics).

**Figure 8:**
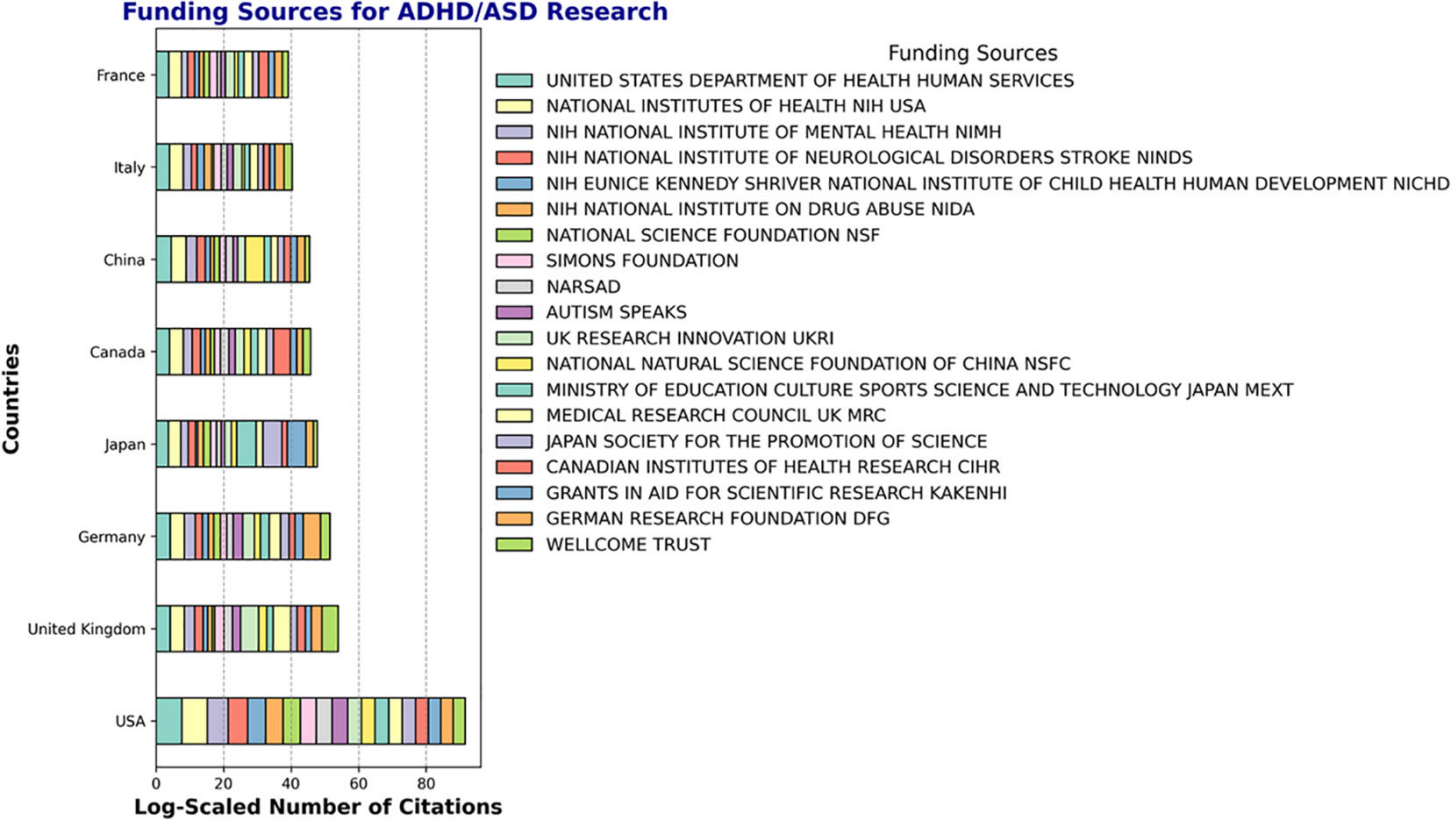
Funding of ADHD/ASD research for the top 8 publishing countries. Data represent the funding sources for each of the top 8 ADHD/ASD research publishing countries (N = 19 funding sources).

## 3 Results

### 3.1 Worldwide publications trends

From 1990 to 2023, a few countries dominated ADHD and ASD research. North America, with the United States (3059 articles), Asia, with China and Japan (487 and 440 publications, respectively), and Europe, with the United Kingdom and Germany (459 and 413, respectively), were among the major contributors. It is worth noting that the United States alone has accounted for more than half of all publications since 1990. Mice and rats were the primary animals used to study ADHD and ASD (**Figure 2**). This is particularly evident in Europe (1801 articles for mice and 1126 for rats), Asia (1079 publications for mice and 622 for rats), and North and South America (2448 publications for mice and 1474 for rats). Of note, Africa and Oceania did not have many publications on ADHD and ASD during the last three decades. Although the data on mice and rats is valuable, it is important to note that in recent years, zebrafish, *Drosophila*, and *C. elegans* have also become increasingly utilized in scientific research. These alternative models offer distinct advantages, such as high-throughput screening capabilities, easier genetic manipulation, and the ability to observe developmental processes in real time, which makes them particularly interesting for studying neurodevelopmental disorders (21, 22). Additionally, 2 papers mentioned the zebrafish in Africa, while 60 papers referenced it in Asia, 128 in Europe, 89 in America, and 10 in Oceania. Since 2000, the number of publications per year for each species has gradually increased, with mice showing the steadiest increase, followed by rats, zebrafish, *Drosophila*, and *C. elegans*.

In summary, we present the worldwide publications on ADHD and ASD from 1990 to 2023, showing a steady increase in research activity over time (**Figure 2**). The use of mice and rats dominated the studies, reflecting their established roles in neurodevelopmental research. While less frequently used, zebrafish, *Drosophila*, and *C. elegans*, provided unique insights into the genetic and developmental aspects of these disorders (12, 23–29).

### 3.2 Geographic distribution of top publishing countries

We next examined the geographic distribution of countries contributing the most to ADHD and ASD research during our investigation timeline. We examined correlations between GDP per capita, life expectancy, and research output (**Supplementary Figures 1**-**5**) because these indicators are often linked to the capacity of a country to invest in scientific research and sustain high publication productivity (30). Higher GDP per capita commonly leads to increased funding for research, while longer life expectancy may be indicative of a well-developed healthcare system, potentially driving biomedical research efforts. For instance, the United States had the highest values for GDP per capita and life expectancy and had the highest publication outputs. Our findings align with previous reports suggesting that countries with higher GDP per capita and life expectancy tend to produce a greater volume of research publications (31). Despite the general trend of higher research output correlating with higher GDP per capita and life expectancy, several European (such as Latvia, Portugal, Belgium) and Asian countries (such as Saudi Arabia, Lebanon) have shown relatively low research output. This could be attributed to various factors, including differences in research prioritization, funding distribution, and institutional support. **Figure 3A** highlights the global distribution of scientific publications related to ADHD and ASD, with America standing out as the primary continent contributing to research, especially in mice, rats, and *Drosophila*. Particularly, the United States consistently leads this research output, followed by other major countries such as China, Japan, the United Kingdom, Germany, Canada, France, and Italy, as shown by the percentage of publications per species for the top 8 countries (**Figure 3B**). Overall, mouse and rat remain the most commonly used model organisms across these nations, with a noticeable trend toward increasing zebrafish and *Drosophila* publications, particularly in countries like the United States and China (**Figures 3A, B**). As shown in **Figure 3C**, the research output in ADHD and ASD has significantly increased over the past decades, particularly in the United States, which saw a sharp rise in publications from 1999 onward, indicating a major shift in research focus for these disorders, although there has been a decrease in trend since 2020. China also experienced a marked increase in research starting around 2008, reflecting growing awareness of ADHD/ASD, and the trend is exponentially rising since. Other countries, such as Japan, Germany, and Canada, began contributing to the literature in the early 2000s, with consistent growth thereafter. The United Kingdom, Italy, and France followed, with their contributions increasing more gradually over the years. Interestingly, analysis of the percentage of ADHD/ASD publications by each country indicated that the United States accounted for more than half of the total publications of the 8 major countries (**Figure 3D**). The other half is shared by the remaining which had less than 10% publication each, principally China, the United Kingdom, Japan and Germany.

In summary, the growing publication trends underline the important role of animal models in addressing ADHD and ASD.

### 3.3 Impact factor and citations

We then examined the distribution of impact factors across journals publishing ADHD and ASD research, noting that high-impact journals are primarily from the United States, Canada, and the United Kingdom (**Figures 4A, B**). Various criteria are used to assess research visibility and influence around the world. Among these, the impact factor, which represents the yearly average number of citations of articles published in the previous two years, and journal citations are commonly employed measures (32). We demonstrate that the top 8 countries published consistently in journals with impact factors (IF) between 2-8 (**Figure 4A**). For journals with IF between 10-20, the number of publications was lower; however, countries like the United States, Germany, France and Canada maintained a standard publication amount. Interestingly, although the number of publications was relatively low (< 10 publications), the top 8 countries have published in journals with IF ranging from 21 to 40, with the United States being the main contributor, followed by China, Italy and France (**Figure 4B**). Furthermore, while the United States and the United Kingdom have published in high IF journals, only Canada did not show publications in journals with IF ranging from 41-60 (**Figure 4B**). For journals with IF between 61-80, the top 8 countries have all contributed, with more publication output from the United Kingdom. While Canada did not have publication count for IF 41-60, it did contribute in IF 81-100, with the United Kingdom and the United States.

We next examined the number of times publications were cited for the top 8 countries and the United States takes the lead anew (**Figures 5A, B**). It is interesting to note that the number of publications had an exponential decrease depending on the number of citations for all countries (the higher the number of citations, the lesser the number of publications). While the USA was always represented for 10–20 times citations, only a few countries like China, Germany and Japan showed inputs (**Figure 5A**). Furthermore, it is worth mentioning that no publication from France was cited more than 9 times. The United States had citations going up to 100 citations, and only Canada, Italy and the United Kingdom were represented with 21–40 citations. China, Germany and Japan had publications with 81–100 citations.

### 3.4 Research focus areas and micro-topics

We then examined the research areas investigated by ADHD and ASD publications. WoS offers easy classification of research areas such as neuroscience, behavioral science, or psychiatry. Interestingly, the United States revealed to be the top country with more than 1500 neuroscience-related publications (**Figure 6**). This was followed by less than 500 publications in neuroscience for the remaining countries, mainly United Kingdom, Germany, China, and Japan. The second major research area is Psychiatry, followed by Pharmacology-Pharmacy, Biochemistry-Molecular Biology, and Behavioral sciences. However, developmental biology accounted for the fewest research areas with few papers on ADHD and ASD. In general, all the countries have publications in the top 10 research areas.

We further investigated the trends for each of the 8 major countries regarding the research topics (**Figure 7**). Topics such as *Autism, Rett Syndrome, ADHD, Deep Brain Stimulation*, and *Oxytocin* exhibit the highest volume of publications across multiple countries. The USA maintains a prominent position in terms of research output across almost all topics, followed by Japan, the United Kingdom, and Germany. Additionally, topics such as *Ultrasonic Vocalization, Prader-Willi Syndrome, Fear Conditioning*, and *Nicotine* show moderate levels of research activity. These topics appear to be gaining interest but still receive comparatively fewer publications than leading research areas. Of particular interest, countries such as China, Canada, and Italy show steady contributions across a broad range of topics, although with lower overall publication volumes compared to the USA and Japan. France maintains a notable presence, particularly in niche research areas, such as *Autism*, *Rett Syndrome*, or *Gambling*. These findings highlight the multifaceted approach researchers are taking to address the complexities of ADHD and ASD.

### 3.5 Funding sources

We next asked about the funding of ADHD and ASD research over the past three decades (**Figure 8**). The United States dominates in terms of the number of funding sources supporting ADHD/ASD research, with major contributions from agencies such as the National Institutes of Health (NIH), the United States Department of Health and Human Services (HHS), the National Science Foundation (NSF) and diverse NIH institutes such as NIMH, NICHD, and NIDA. Moreover, multiple countries, including Japan, Germany, the United Kingdom, and Canada, have diverse funding sources supporting this research. In the United Kingdom, the Medical Research Council (MRC) and UK Research Innovation (UKRI) play important roles. Japan receives funding primarily from the Ministry of Education, Culture, Sports, Science, and Technology (MEXT) and the Japan Society for the Promotion of Science (JSPS). European countries, including France, Germany, and Italy, receive support from agencies such as the German Research Foundation (DFG) and the Wellcome Trust, and China receives significant funding from the National Natural Science Foundation of China (NSFC). While the USA contributes significantly to the research landscape, other countries demonstrate balanced and distributed funding efforts, supporting a global focus on ADHD/ASD research. The number of publications that have acknowledgments for funding societies are presented in detail for Europe (**Supplementary Figure 6**), Asia (**Supplementary Figure 7**) and North America (**Supplementary Figure 8**). This analysis underscores the importance of funding in driving research activity and highlights the collaborative nature of ADHD and ASD research across different regions.

### 3.6 Network analysis

Last, we investigated the network of ADHD and ASD publications and utilized the data exported from WoS for analysis using VOS viewer version 1.6.20. We identified 17 clusters with the United States as the principal central node. For example, cluster 5 included the United States, Colombia, Ghana, Saint Vincent and Turkey. We present a VOS viewer network analysis illustrating the collaborations and research impact among all countries worldwide from 1990 to 2023 (**Supplementary Figure 9**). Additionally, to better visualize the network’s importance, we provide a heatmap view, indicating the intensity of nodes for each country’s power in the network (**Supplementary Figure 10**). We next represented the average number of citations per year for each country within the network, demonstrating that the United States, followed by China, Germany and the United Kingdom, dominated the citation count of ADHD and ASD research (**Supplementary Figure 11**). However, research in ADHD and ASD in the United States appears to have slowed in the late 2020s (**Supplementary Figure 12**), with fewer publications. Interestingly, smaller nodes such as Sweden, Turkey, Portugal, or Ghana, for example, have shown recent interest in the topics and have begun publishing ADHD/ASD-related research. This means that although the prevalence is not increasing in these countries, research interest has been growing.

In summary, the comprehensive analysis of ADHD and ASD-related papers from 1990 to 2023 identifies key trends, influential countries, and collaborative networks. The relationship between research activity and disease prevalence shows that countries with larger research outputs are likewise more impacted by these disorders. The use of numerous animal models has helped advance our understanding of ADHD and ASD, resulting in better diagnosis, treatment, and management. Continued worldwide collaboration and diverse approaches are required to address the complex nature of these neurodevelopmental disorders and make significant advances in this crucial area of research.

## 4 Discussion

This comprehensive analysis of ADHD and ASD-related papers from 1990 to 2023 identifies numerous major patterns, including the dominant contributions of specific countries and shifting research approaches in the field of neurodevelopmental disorders. The United States has emerged as the dominant contributor to ADHD and ASD research, accounting for more than half of all publications since 1990. This dominance can be linked to a strong research infrastructure, and significant funding from agencies such as the NIH and NIMH (33). Europe and Asia, namely the United Kingdom, Germany, China, and Japan, make substantial contributions to the research landscape. Furthermore, the relationship between GDP per capita, life expectancy, and research output indicates that wealthier countries with longer life expectancies contribute more in scientific research. However, other metrics such as population number, disease prevalence, research interest, and policy agendas, can have a large impact in the number of output publications. For example, China’s increasing research output since 2008 could reflect greater government expenditure in scientific research and growing awareness of neurodevelopmental disorders (34).

Mice and rats have been the most employed species in ADHD and ASD research on all major continents, reflecting their established roles as model organisms in neuroscience (11, 35). These models are extremely useful for studying the genetic, molecular, and behavioral components of disease. The growing use of alternative models such as the zebrafish, *Drosophila*, and *C. elegans* demonstrates a trend toward using varied models to generate novel insights, particularly in genetic and developmental studies. Thus, the progressive growth in publications using zebrafish, *Drosophila*, and *C. elegans* since the early 2000s indicates a rising appreciation for their utility in high-throughput screening and ability to simulate important aspects of neurodevelopmental disorders (12, 23–29). This trend underscores the importance of expanding the repertoire of animal models to enhance our understanding of ADHD and ASD. Furthermore, our analysis reveals that research areas are predominantly focused on neuroscience, molecular biology and behavioral sciences, reflecting the topics related to core features of ADHD and ASD (36). On the other hand, developmental biology remains underrepresented, indicating a potential gap in understanding the early developmental processes underlying these disorders. The emphasis on genetics, pharmaceutical therapies, and neuroimaging techniques demonstrates the broad approach that researchers are using to address the complexity of ADHD and ASD.

High-impact journals and citation analysis demonstrate the impact of research from the United States, Canada, and the United Kingdom. The constancy of impact factors among the top publishing countries indicates that research quality is largely consistent, emphasizing the global aspect of scientific research. Finally, funding is crucial in driving research activity, with key research funders such as the NIH, NIMH, and MEXT making significant contributions to their respective countries’ high publication output. Furthermore, the network analysis demonstrates the collaborative character of ADHD and ASD research, with substantial collaboration between countries, particularly those with established research infrastructures. The establishment of new research nodes in Sweden, Turkey, Portugal, and Ghana demonstrates an increasing worldwide interest in ADHD and ASD from various regions of the world. This trend is encouraging since it indicates growing awareness and the potential for fresh contributions from other geographic regions.

The steady increase in ADHD and ASD research publications over the past three decades indicates significant progress in the field. However, there are specific points that require further attention. Notably, additional study is required to better understand the early developmental stages of ADHD and ASD, which could lead to earlier diagnosis and prevention. Similarly, we believe that continued diversification of animal models will reveal more information on the genetic and molecular pathways that underpin these disorders. Strengthening international cooperation and offering research support to underrepresented regions like Africa, can improve research output of ADHD and ASD. Nonetheless, Africa and Oceania’s limited contribution to ASD and ADHD research does not imply that researchers from these regions are uninterested in the topic. It is possible that scientists from Africa and Oceania are contributing to significant research in countries providing more resources and funding, such as the United States, Japan, and China. This underlines the importance of increasing the visibility of underrepresented regions by raising awareness, providing research funding, and adequate infrastructure. Sustained and expanded support from both government and non-governmental sources will be critical for furthering research and translating discoveries into clinical practice.

## 5 Conclusion

In conclusion, our findings highlight the interesting dynamic and developing nature of ADHD and ASD research. The scientific community can continue to make considerable progress in understanding and treating these complex neurodevelopmental disorders by leveraging varied animal models, increasing international collaboration, and resolving research gaps.

## 6 Limitations of the study

Our study has limitations, as per the words/terms inclusion criteria in the search strategy. Some terms previously used in ASD research/diagnostic like “Infantile autism”, “Asperger syndrome”, “Asperger disorder”, “Childhood disintegrative disorder”, “Heller’s syndrome”, “Disintegrative psychosis” and “Pervasive developmental disorder”, were not considered in this report. Similarly, “Hyperkinetic disorder” was not included in the search strategy for ADHD. It is important to mention that this diagnostic term is considered a more severe form, with excessive, uncontrollable hyperactivity and impulsiveness, often overlapping with ADHD.

## Data Availability

The original contributions presented in the study are included in the article/Supplementary Material, further inquiries can be directed to the corresponding author/s.
