# Supplementary material for: Three decades of neuroscience research using animal models of ADHD and ASD: a bibliometric analysis
Source: Front Psychiatry. 2025 May 19;16:1528205. doi: 10.3389/fpsyt.2025.1528205 (PMC12127757; doi:10.3389/fpsyt.2025.1528205)
Supplement: Supplementary file 1 [file DataSheet1.pdf]

Supplementary Material

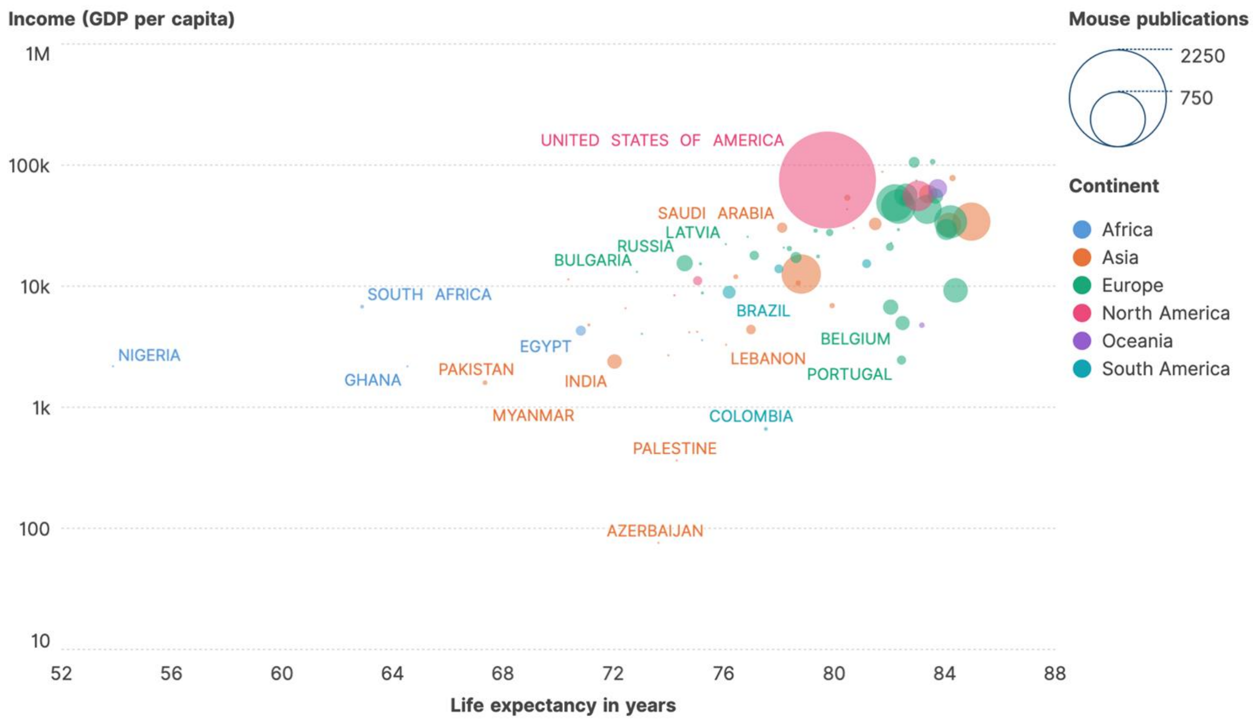

Supplemental Figure 1. Scatter plot of mouse total publications per countries depending on life expectancy and GDP per capita. The plot shows a graphical view of publication trends for mouse in the world.

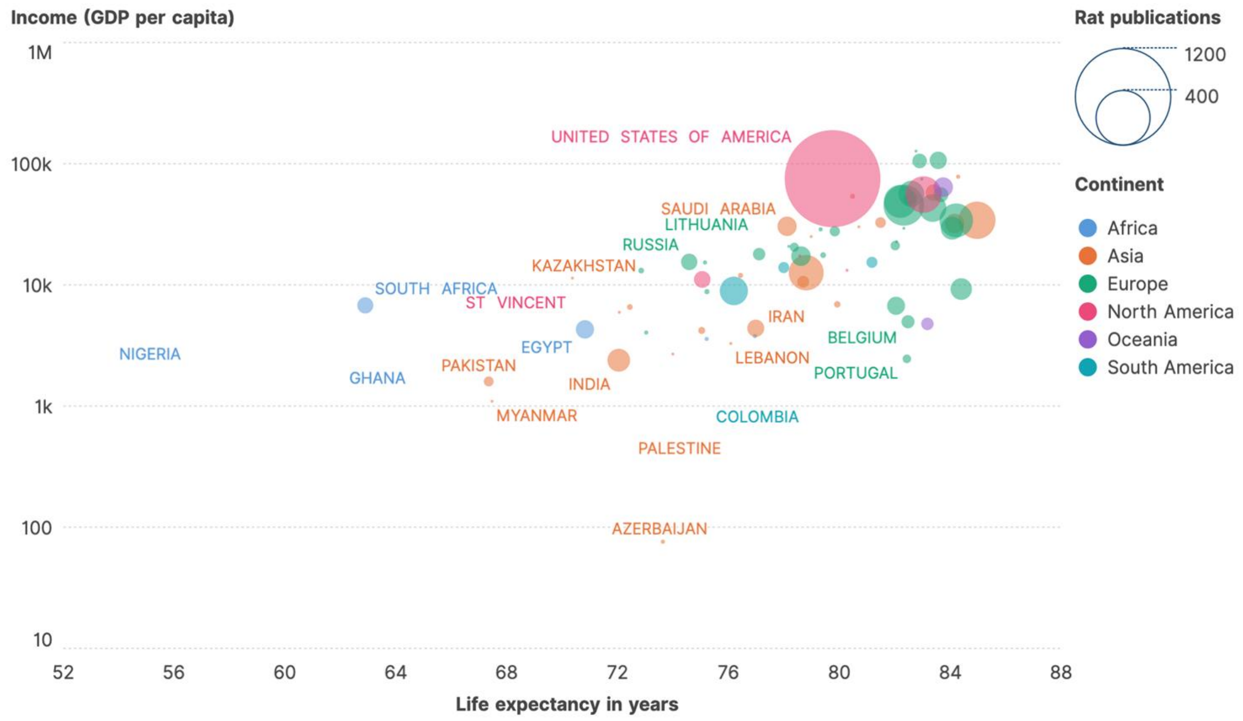

Supplemental Figure 2. Scatter plot of rat total publications per countries depending on life expectancy and GDP per capita. The plot shows a graphical view of publication trends for rat in the world.

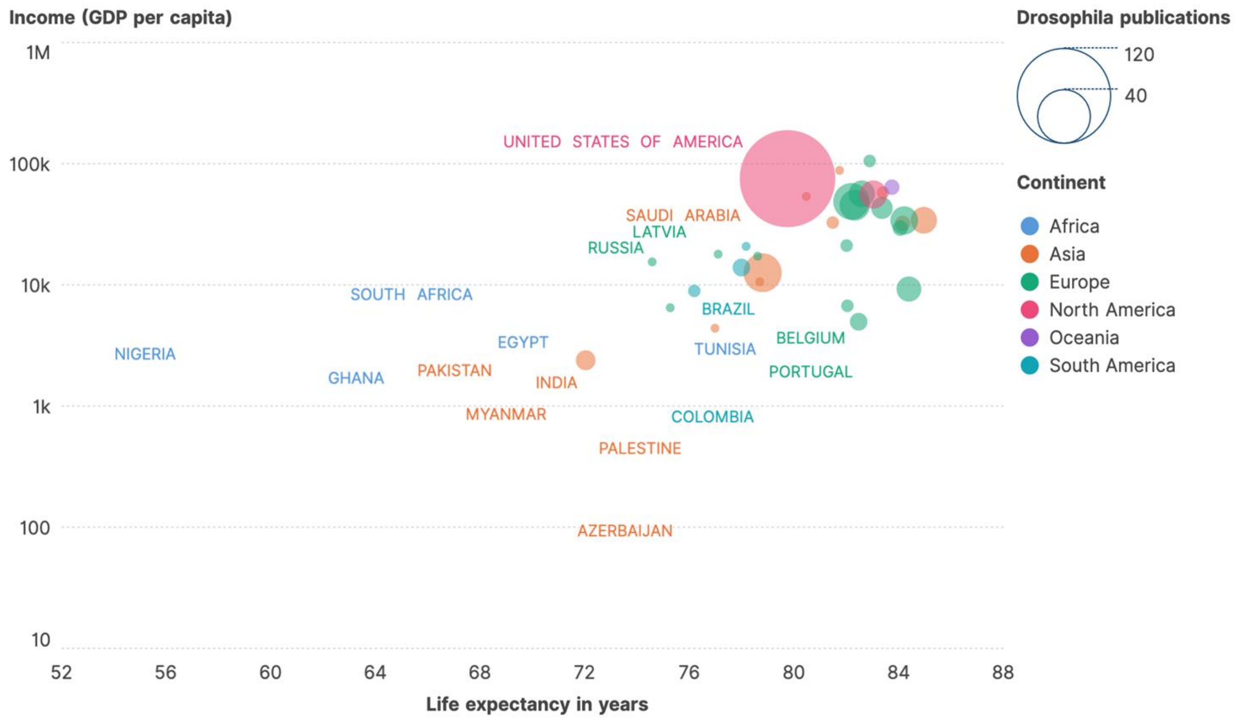

Supplemental Figure 3. Scatter plot of Drosophila total publications per countries depending on life expectancy and GDP per capita. The plot shows a better graphical view of publication trends for Drosophila in the world.

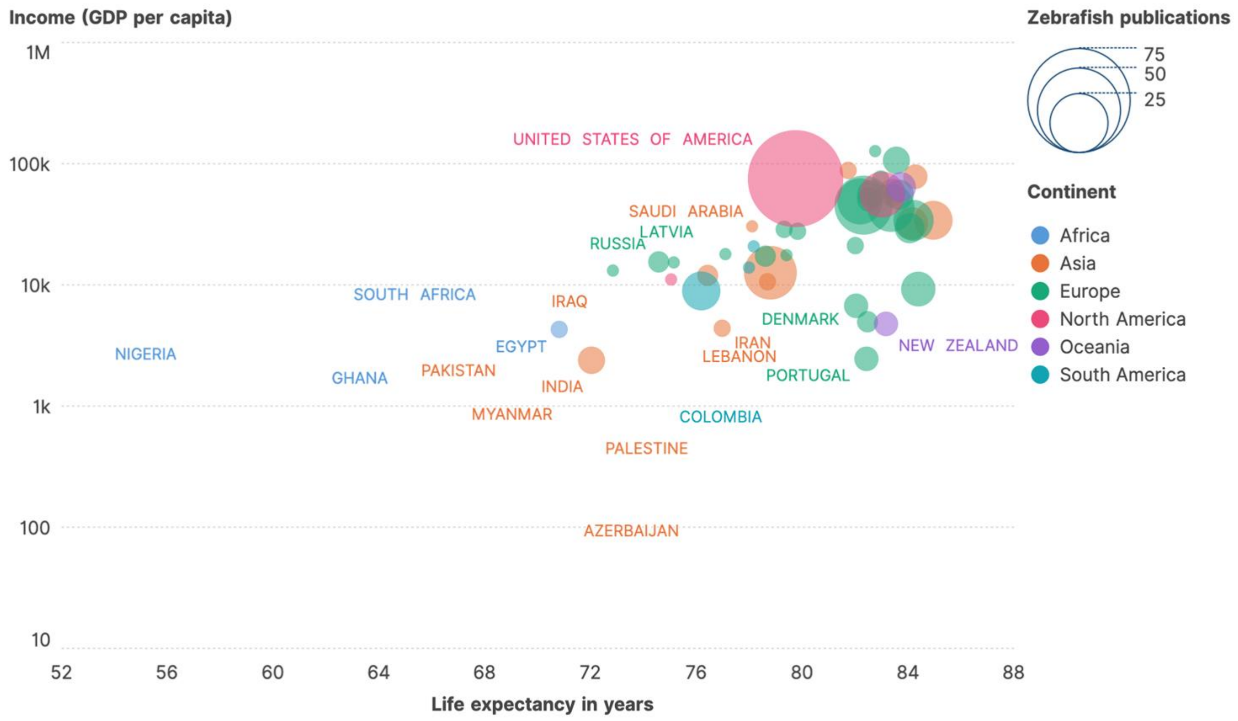

Supplemental Figure 4. Scatter plot of zebrafish total publications per countries depending on life expectancy and GDP per capita. The plot shows a better graphical view of publication trends for zebrafish in the world.

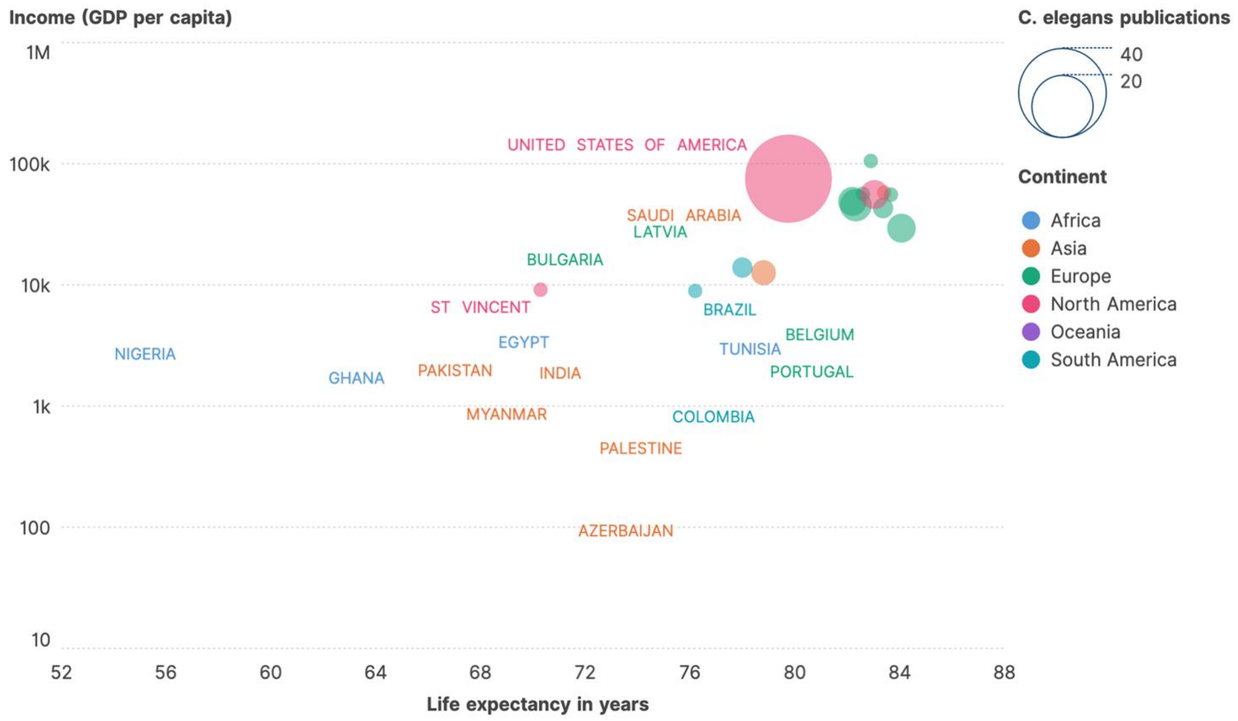

Supplemental Figure 5. Scatter plot of C. elegans total publications per countries depending on life expectancy and GDP per capita. The plot shows a better graphical view of publication trends for C. elegans in the world.

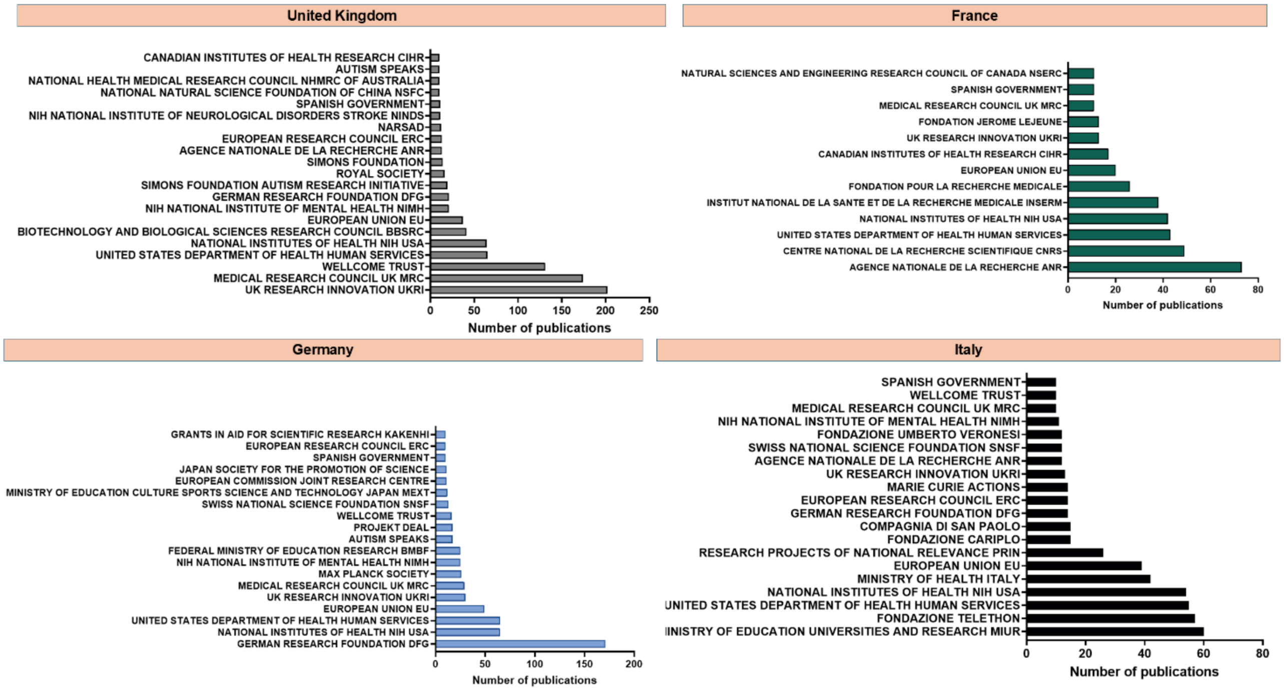

Supplemental Figure 6. Top funders of ADHD/ASD research in Europe. Each country is represented with the top funders mentioned.

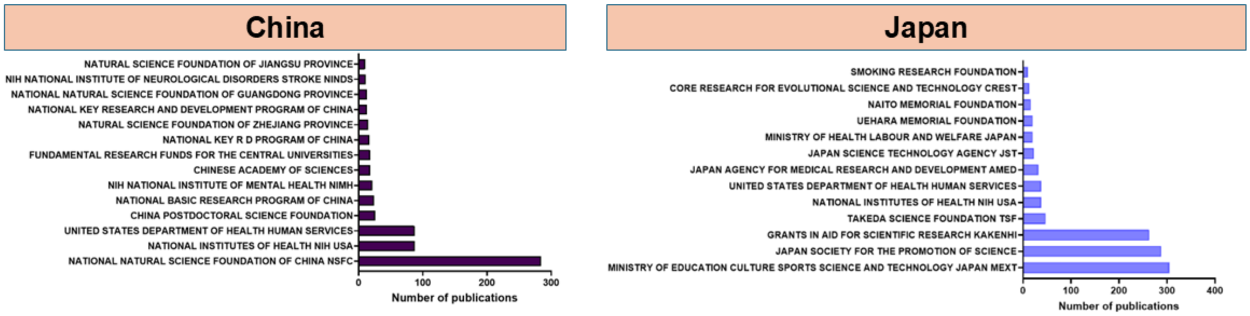

Supplemental Figure 7. Top funders of ADHD/ASD research in Asia. Each country is represented with the top funders mentioned.

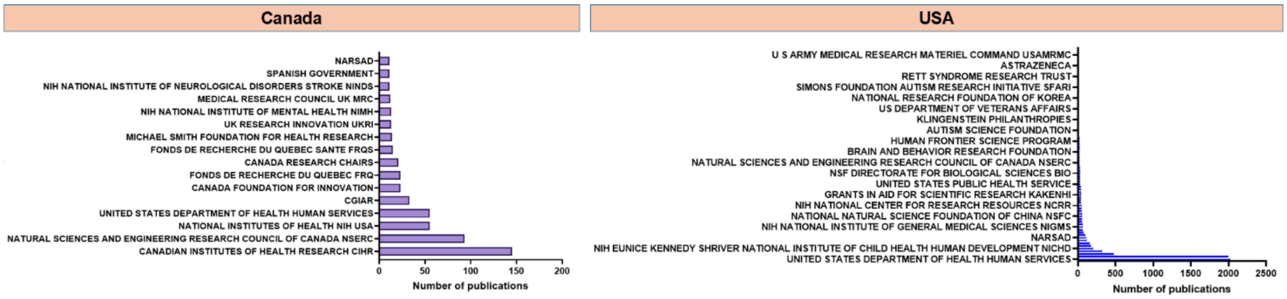

Supplemental Figure 8. Top funders of ADHD/ASD research in North America. Each country is represented with the top funders mentioned.

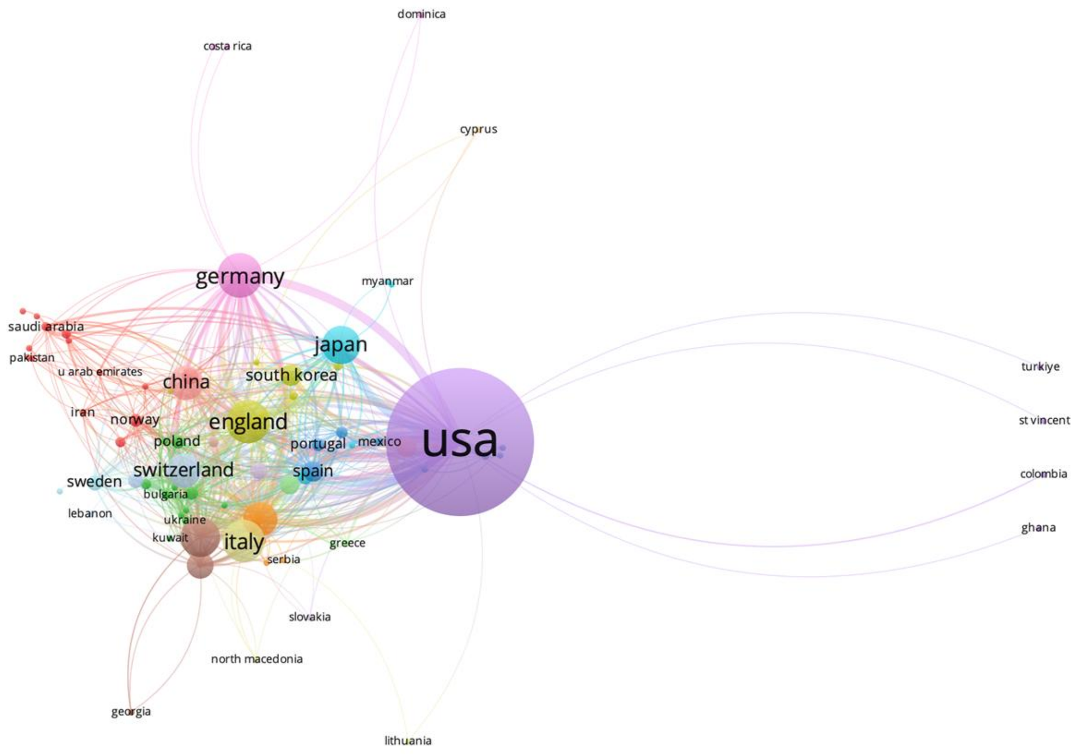

Supplemental Figure 9. Network view of countries/regions contributing to ADHD and ASD research. 17 clusters are indicated, with the United States representing the major node among countries.

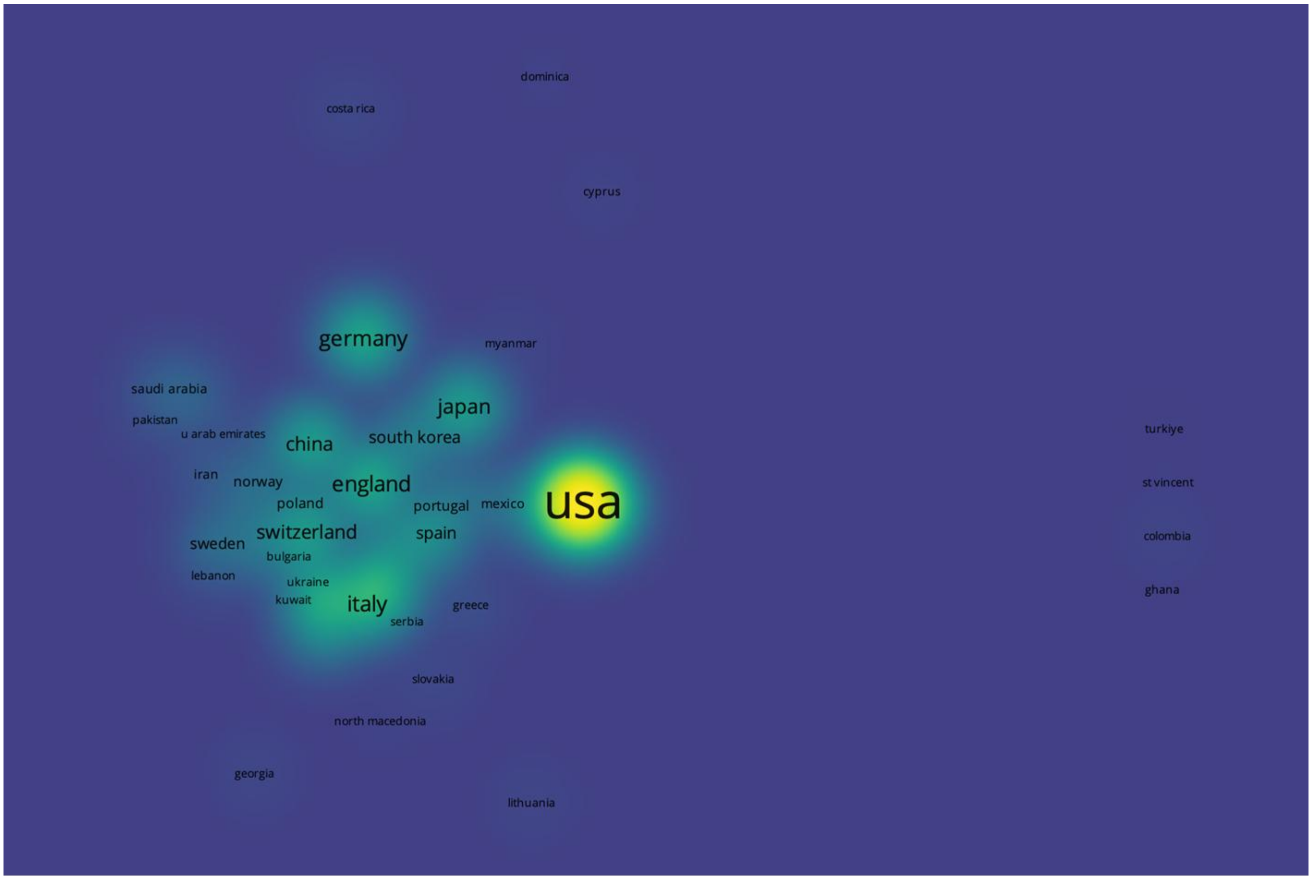

Supplemental Figure 10. Network view of countries/regions contributing to ADHD and ASD research (density view). 17 clusters are indicated, with the United States representing the major node among countries.

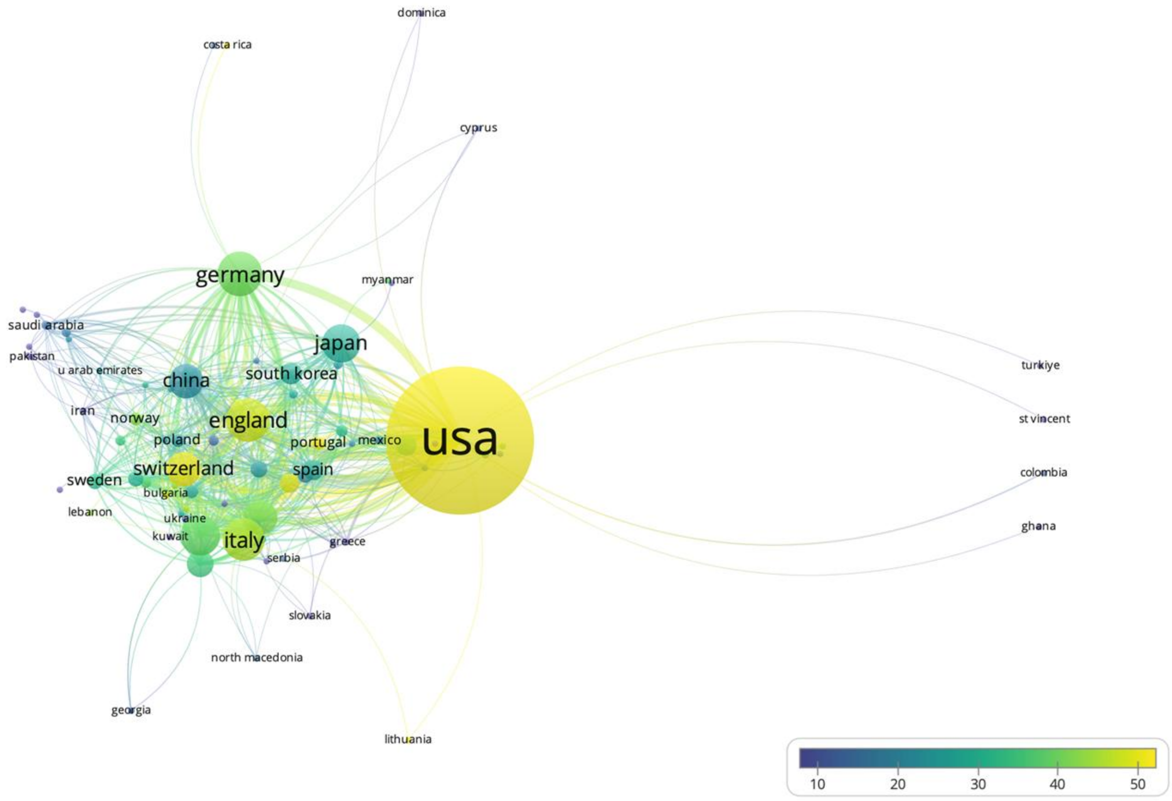

Supplemental Figure 11. Network view of countries/regions contributing to ADHD and ASD research. Data represents average citations per countries as indicated by the intensity gradient.

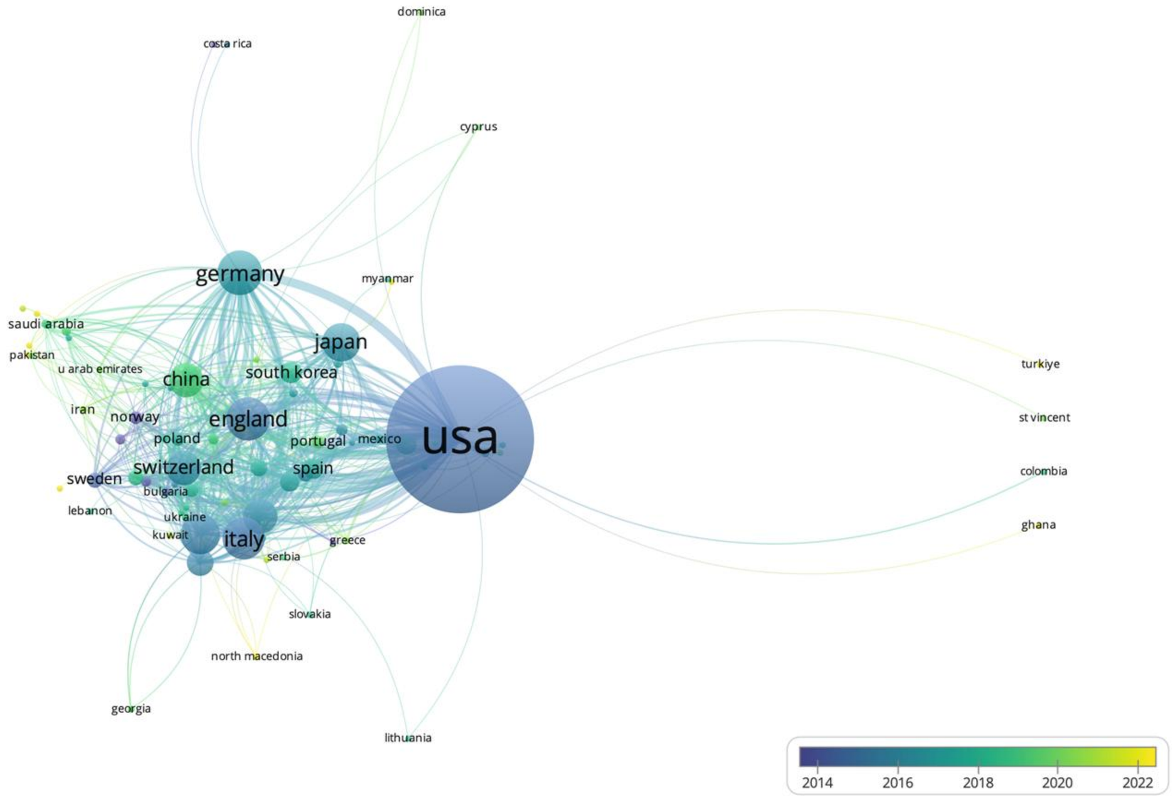

Supplemental Figure 12. Network view of countries/regions contributing to ADHD and ASD research. Data represents number of publications per year per countries as indicated by the intensity gradient.
